# Premature Attraction of Pollinators to Inaccessible Figs of *Ficus altissima*: A Search for Ecological and Evolutionary Consequences

**DOI:** 10.1371/journal.pone.0086735

**Published:** 2014-01-22

**Authors:** Yuan Zhang, Yan-Qiong Peng, Stephen G. Compton, Da-Rong Yang

**Affiliations:** 1 Key Laboratory of Tropical Forest Ecology, Xishuangbanna Tropical Botanical Garden, Chinese Academy of Sciences, Kunming, China; 2 Yunnan Academy of Biodiversity, Southwest Forestry University, Kunming China; 3 School of Biology, University of Leeds, Leeds, United Kingdom; 4 Department of Zoology and Entomology, Rhodes University, Grahamstown, South Africa; Ghent University, Belgium

## Abstract

Adult life spans of only one or two days characterise life cycles of the fig wasps (Agaonidae) that pollinate fig trees (*Ficus* spp., Moraceae). Selection is expected to favour traits that maximise the value of the timing of encounters between such mutualistic partners, and fig wasps are usually only attracted to their hosts by species- and developmental-stage specific volatiles released from figs at the time when they are ready to be entered, oviposited in and pollinated. We found that *Ficus altissima* is exceptional, because it has persistent tight-fitting bud covers that prevent its *Eupristina altissima* pollinator (and a second species of ‘cheater’ agaonid) from entering its figs for several days after they start to be attracted. We examined the consequences of delayed entry for the figs and fig wasps and tested whether delayed entry has been selected to increase adult longevity. We found that older pollinators produced fewer and smaller offspring, but seed production was more efficient. Pollinator offspring ratios also varied depending on the age of figs they entered. The two agaonids from *F*. *altissima* lived slightly longer than six congeners associated with typical figs, but this was explainable by their larger body sizes. Delayed entry generates reproductive costs, especially for the pollinator. This opens an interesting perspective on the coevolution of figs and their pollinators and on the nature of mutualistic interactions in general.

## Introduction

Maximum life spans among the estimated 750,000 described species of insects differ over 5000-fold, and adult life spans vary from just one or two days in mayflies and fig wasps to several years in the case of some ant and termite queens [1]. Maximum life spans also vary within species, most obviously in social insects, with honeybee queens capable of living 3–5 years, far longer than drones or workers [2]. The relative lengths of the adult and larval stages in insects broadly reflect relative resource acquisition of the two life history stages, with extended larval durations exhibited by species such as cicadas that feed on low quality diets and the shortest adult life spans exhibited by species that do not feed at all as adults and depend entirely on the resources obtained as larvae for their metabolic and reproductive needs [3], [4]. Although a fundamental trait of insect species, longevity can be responsive to selection [5], [6]. Realized longevity can also be correlated with body size, in part because larger individuals can display greater resistance to environmental stresses such as dehydration [7]–[10]. Selection acting on longevity and body size are therefore not necessarily independent.

*Ficus* (Moraceae) is a pantropical genus containing over 750 species that is of great ecological significance because of the large numbers of animals that feed on figs, also known as syconia [11]. Figs are closed, urn-shaped infloresences lined with tiny uniovulate female flowers that are pollinated exclusively by tiny fig wasps (Agaonidae). Adult female fig wasps enter receptive figs through a narrow slit-like ostiole in order to oviposit in the female flowers. They also bring with them pollen from their natal figs [12], [13]. Pollinator offspring develop in galled ovules, completing their development at about the time that the seeds have also matured [14]. After mating, adult females of the next generation emerge through an exit hole in the fig wall created by their males and fly in search of receptive figs, which will usually be on another tree [15]. Adult pollinator fig wasps are pro-ovigenic, emerging with a full complement of mature eggs and do not feed as adults [16], [17]. Adult male fig wasps are equally short-lived, and most spend their adult lives within their natal figs. Mature figs produced by monoecious fig trees each contain a mixture of seeds and pollinator offspring, together with the offspring of non-pollinating fig wasps (NPFW) that have developed at the expense of the plant’s ovules or pollinator offspring. Most NPFW lay their eggs from the outside of the figs, and do not need to penetrate the ostiole.

Fig wasps develop inside galled ovules, the size of which is correlated with their adult size. Females of different agaonid species vary in length from less than one mm to about 3 mm [18]. Stabilizing selection is likely to be determining on their body sizes because head shape and maximum body size are strongly constrained by the shape and size of the ostioles through which they must crawl to reach their oviposition sites within receptive figs [19]. This is because larger individuals are more likely to become trapped when attempting entry [20], [21]. Conversely, larger individuals are more likely to successful reach receptive figs, suggesting that they may live longer [20], [21]. Whether adults of larger fig wasp species also live longer than those of smaller species has not been confirmed.

In obligate mutualistic systems such as that involving figs and fig wasps, selection generated by one partner elicits morphological and behavioral responses in the other, leading to their co-evolution. Selection is also expected to favor traits that maximize the value of the timing of encounters between mutualistic partners, especially when it must be repeated at each generation [22], [23]. The importance of timing is particularly apparent in the case of pollination of fig trees, because each *Ficus* species is pollinated exclusively by one or a small number of host specific species of fig wasps [24], [25], the adults of which have very short life spans of typically one or two days [13], [26], [27]. Consequently, pollinators that are attracted too soon are likely to die before they can pollinate the plant, and pollinators attracted after the figs are no longer receptive have little time for flight to other trees with suitable figs, which are often at low densities [28], [29]. Figs that have to wait to be pollinated are less productive [23], but whether fig wasps that are older when they enter figs are less effective pollinators, or less fecund is less clear.

To ensure synchronization of fig wasp attraction with the presence of figs that are ready to be entered, figs emit developmental stage and species-specific volatiles that are only attractive to their particular species of pollinators at the time when the figs are receptive [30]–[32]. The duration of receptivity is prolonged if a fig remains unpollinated [22], [23], [33], which means that receptive figs can ‘wait’ for pollinators if they are in short supply at the time when they first become receptive. Conversely, attractant volatile production ceases, and the ostiole closes, after pollinators enter the figs [30], [31], [34], [35].

**Figure 1:**
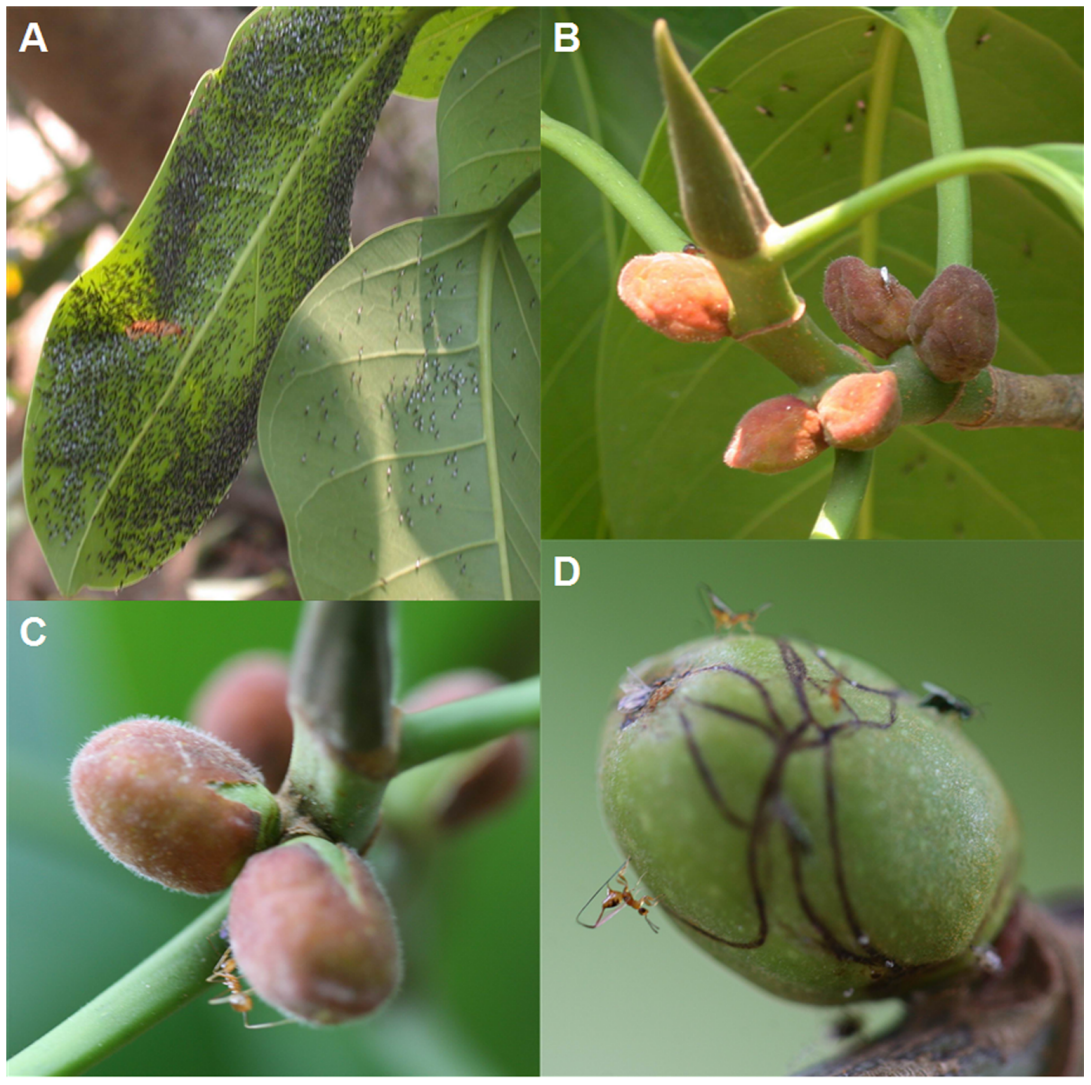
Anomalous phenomenon about early arrival of pollinators and entering the receptive figs. (a) *Eupristina altissima* and *Eupristina* sp. females clustering beneath leaves of *F*. *altissima*; (b) Pre-receptive figs of *F*. *altissima* entirely covered by persistent bracts; (c) Older figs where the bracts have started to detach, allowing entry to the ostiole; (d) A fig after pollination, with surface tracks indicating the routes that agaonid females had taken when crawling beneath the bracts. Also NPFW females ovipositing from the fig surface.

Given the importance of ensuring synchrony of fig wasp attraction with immediate access to receptive figs, the discovery of a fig tree that attracts pollinators before they can enter its figs would be surprising, but this appears to be the case with the Asian species *F*. *altissima*. The figs of this species are unusual in that they retain large enveloping bracts until the time they are pollinated. A large *F*. *altissima* individual at Xishuangbanna (XTBG), in South China was monitored for 10 years. During this period it produced two unequal-sized flushes of new leaves and two crops of new figs each year (Y-Q Peng, unpublished). Generally, the tree shed all of its leaves once a year and then initiated new leaves and numerous figs simultaneously. The second periods of leaf loss each year were less extensive, and were followed by small quantities of new leaves and new figs that appeared on associated branch tips. Agaonids were seen to arrive at the tree at times when it was bearing large crops of immature figs, forming large aggregations on the underside of the young leaves (Figure 1a). At this time the figs were unsuitable for entry, because they were still enclosed by persistant bracts that surround the figs and prevent entry into the ostiole (Figure1b). Pollinators were only seen to enter the figs once the figs became more rounded and the surrounding bracts split apart (Figure 1c). The female fig wasps could then crawl towards the ostiole through the narrow space available between the fig wall and bracts, often leaving marks on the fig wall that indicated the routes they had taken that become visible once the bracts become detached (Figure 1d). After the bracts had fallen from the figs they became accessible to NPFW that lay their eggs from the outside of figs and destroy pollinator larvae or gall ovules (Figure 1d). Less intense observations of other *F. altissima* individuals confirmed that ‘early’ arrival of pollinators is typical for this species.

Here we (1) characterise the relationship between the timing of fig wasp arrivals and the developmental states of *F*. *altissima* figs, (2) examine ecological consequences of a mis-match between pollinator attraction and pollinator entry by recording the reproductive success of the figs and pollinators in relation to pollinator and fig age and (3) determine whether premature attraction to trees before their figs can be entered has selected for extended longevity among the agaonids associated with the tree.

## Materials and Methods

### Study Species and Sites

The study was carried out at Xishuangbanna Tropical Botanical Garden (XTBG), located in tropical Southwest China (21°55′ N, 101°15′ E, at about 555 m ASL). No specific permissions were required for these locations/activities. Because the location is not privately-owned or protected in any way, and field studies did not involve endangered or protected species.

*Ficus altissima* Blume (subgenus Urostigma, section Conosycea) is a monoecious fig tree species distributed across Asia [36], [37]. It occurs naturally in tropical forest at Xishuangbanna and is also frequently planted in cities and villages or near temples as an ornamental or sacred plant. The population size of *F. altissima* is large in the Xishuangbanna region. Leaf production of *F*. *altissima* occurs at irregular intervals throughout the year, with new leaves and figs (syconia) initiated together. The figs of *F. altissima* are axillary and paired (rarely solitary), reaching about 15 mm when mature. They are produced in synchronous crops, but with asynchrony between trees [38]. Large crops can number many thousands. *Ficus altissima* is actively pollinated by the agaonid *Euptistina altissima*, but its figs also support an undescribed congener (‘cheater’ *Eupristina* sp.) which has reduced pollen pockets and fails to pollinate [39], but enters figs at the same developmental stage. This phenology, with synchrony within the trees and asynchrony between trees ensures the agaonid wasps have a chance to find receptive figs. The volatile chemicals that attract the two *Eupristina* species have not been characterised.

The onset of receptivity of the figs of most *Ficus* species usually corresponds with the opening of their ostioles, which allows attractant volatiles to be released from inside the figs and female pollinators to penetrate them. In contrast, the point at which the figs of *F*. *altissima* become receptive and accessible to pollinators is determined by a combination of ostiolar opening and a loosening of the surrounding bracts. To avoid confusion, we use the term ‘accessible’ to describe the period when *F*. *altissima* figs can be entered by pollinators, because the onset of receptivity as defined in other fig species may have taken place some time before the agaonids associated with *F*. *altissima* are physically able the enter the figs.

### Fig Wasp Arrivals at a Receptive Tree

A tree with young developing figs was monitored daily. Once the first agaonid was observed, ten sticky traps (yellow flat sheets, 21×15 cm) were suspended from the lower branches, 2 m above the ground around the tree, and monitored daily from the time when the first fig wasps appeared. The sticky traps were replaced daily, at 8∶00 AM, and the numbers of the two *Eupristina* species were recorded. Trapping continued for 30 days, by which time no new pollinators wasps were recorded.

### Experimental Introductions of Fig Wasps of Varying Ages

Young pre-receptive figs were enclosed in nylon bags to prevent oviposition by pollinators and other fig wasps. The figs became accessible to pollinators once the surrounding bracts began to split apart and separate from the figs they had been enclosing. Single female *E*. *altissima* of varying ages were introduced into the figs on the first day that they became accessible. The ability to pollinate and the number and size of the offspring of freshly-emerged individuals were compared with that of older females that had been placed in an incubator maintained at 15°C with a 12∶12. L:D cycle and 80% humidity for 24 hours. The nylon bags were replaced after entry of the pollinators and retained until the almost-mature figs were removed from the trees. The numbers of seeds and pollinator offspring in each fig were counted and the head sizes of 10 female pollinators from each fig were measured to provide an indication of body size (see below for methods).

### Experimental Introductions into *E*. *altissima* Figs of Varying Ages

The effects of fig age at the time of pollinator entry (measured from the onset of accessibility) on the reproductive success of the plant and its pollinator were determined by introducing single freshly-emerged pollinators into figs that had been accessible for varying lengths of time. The figs were bagged as before, and the onset of accessibility was also determined as before. Figs were deemed no longer receptive when three different fig wasps failed to attempt entry. After several weeks, the numbers of mature seeds and pollinator offspring inside each mature fig were also counted and within each fig age group, the head sizes of 10 female pollinators from each of 10 figs were measured as before.

### Adult Female Longevity and Size

The longevity of adult females of eight *Eupristina* species was compared, together with the relationship between body size and longevity. The additional species were *E. koningsbergeri* (from *F*. *benjamina*), *E*. *verticillata* (*F*. *microcarpa*), *Eupristina* sp.1 (*F*. *glaberrima)*, *Eupristina* sp.2 (*F*. *pisocarpa*), *Eupristina* sp.3 (*F*. *maclellandii*) and *Eupristina* sp.4 (*F*. *curtipes*).

Mature figs lacking exit holes, but with agaonid females that had emerged from their natal galls into the lumen of the figs, were collected from their respective tree species. These figs were easily identified by the softening of the fig walls. Groups of 12–50 wasps were placed in clear glass vials with mesh lids that provided ventilation and prevented the wasps from escaping. The vials were stored in an incubator, with temperature set at 25°C with L:D = 12∶12. The numbers of dead fig wasps were recorded every 8 hours. Total sample sizes were 278 (*E*. *altissima*), 367 (‘cheater’ *Eupristina* sp.), 295 (*E. koningsbergeri*), 170 (*E*. *verticillata*), 303, 168, 310 and 176 (*Eupristina* spp. 1–4, respectively).

Head size has been shown to provide a good estimate of overall body size in agaonids [27]. Mature figs containing the eight *Eupristina* species were collected from their respective host trees. Ten figs (lacking exit holes) containing each species were placed separately in nylon bags to allow the adult female fig wasps to emerge. Freshly-emerged individuals were stored in 80% ethanol with a little added glycerine (to reduce collapse of their bodies). Body size estimates were based on measurements of maximum head length and maximum head width, using an eyepiece graticule mounted on an Olympus SZX12 binocular microscope. Five females per fig were measured, giving a total of 50 measurements per species.

### Data Analysis

Paired-samples T tests were used to compare daily differences in the numbers of *E. altissima* and ‘cheater’ *Eupristina* sp. captured on the sticky traps. The differences between seeds numbers and offspring numbers were also compared using paired-samples T tests in the fig/wasp aging experiments. A Cox regression assuming proportional hazards was used to test for between-species differences in wasp longevity, with species cohort as a factor. Censoring was not required because the total life span of all wasps were known. Wasp body size was not included in our survival analysis, because it was impractical to obtain longevity and body size data for all wasps. As an alternative, we used mean longevity of each species as the response variable in a linear model (LM) [27], [40].

The effects of pollinator age on seed and fig wasp offspring production and offspring body sizes were compared using ANOVA. GLMs with Poisson errors were used to analyze the effects of fig age on seed and wasp offspring production, and an LM for the relationship between the age of figs at pollination and body sizes of the agaonid offspring.

All analyses were conducted using R.11.1.

## Results

### Fig Wasp Arrivals at a Receptive Tree

**Figure 2:**
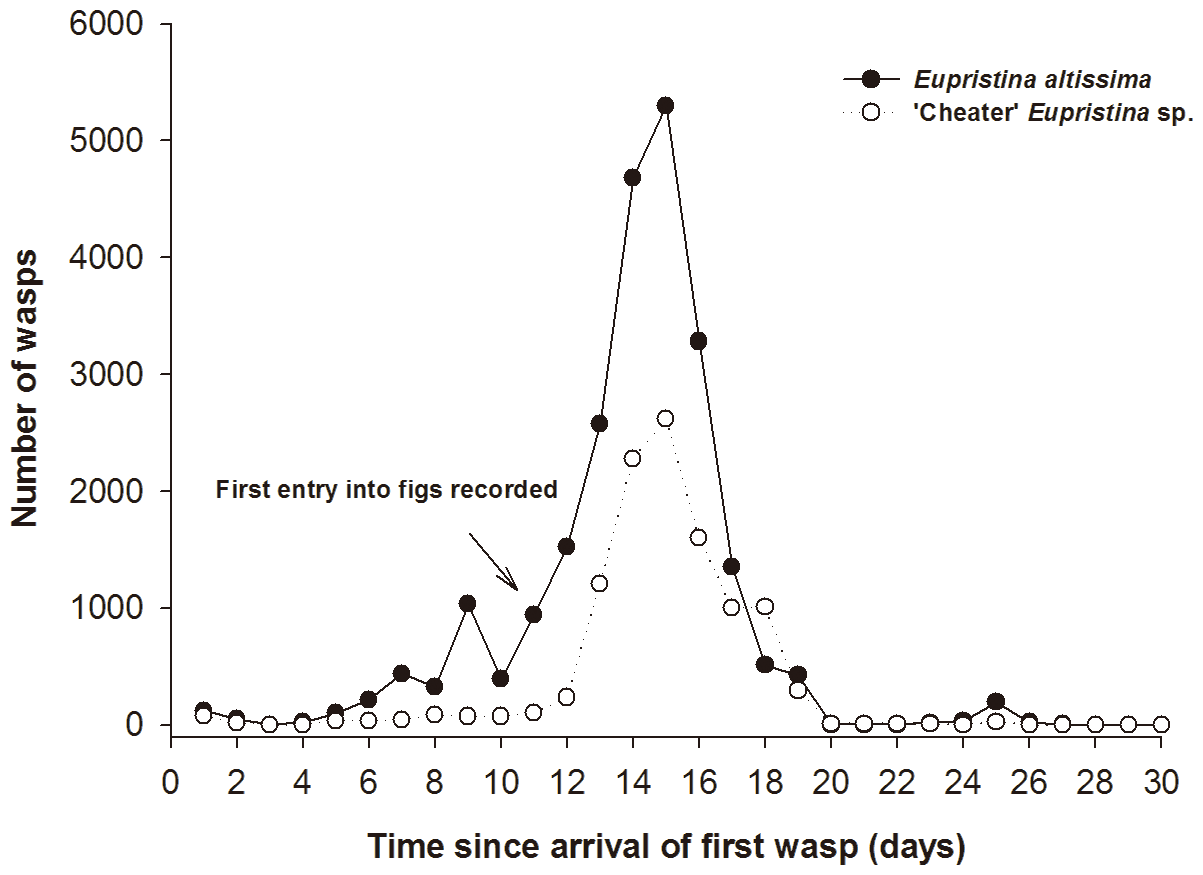
The numbers of agaonid fig wasps trapped at a *F*. *altissima* tree in relation to when the figs became accessible for entry.

Adult agaonids were present on the sticky traps placed around *F*. *altissima* for a 30 day period (Figure 2). The pollinator *E*. *altissima* and cheater *Eupristina* sp. displayed similar patterns of abundance, with the first individuals recorded about 10 days before any of the figs became accessible and their numbers peaking five days after, before declining to zero once all the figs had been entered. During the period before any of the figs were accessible the fig wasps accumulated on nearby young leaves. Pollinators were more frequently trapped than cheaters (paired-samples t-test: *t*_29_ = 3.10, *p*<0.05).

### Experimental Introductions of Fig Wasps of Varying Age

**Figure 3:**
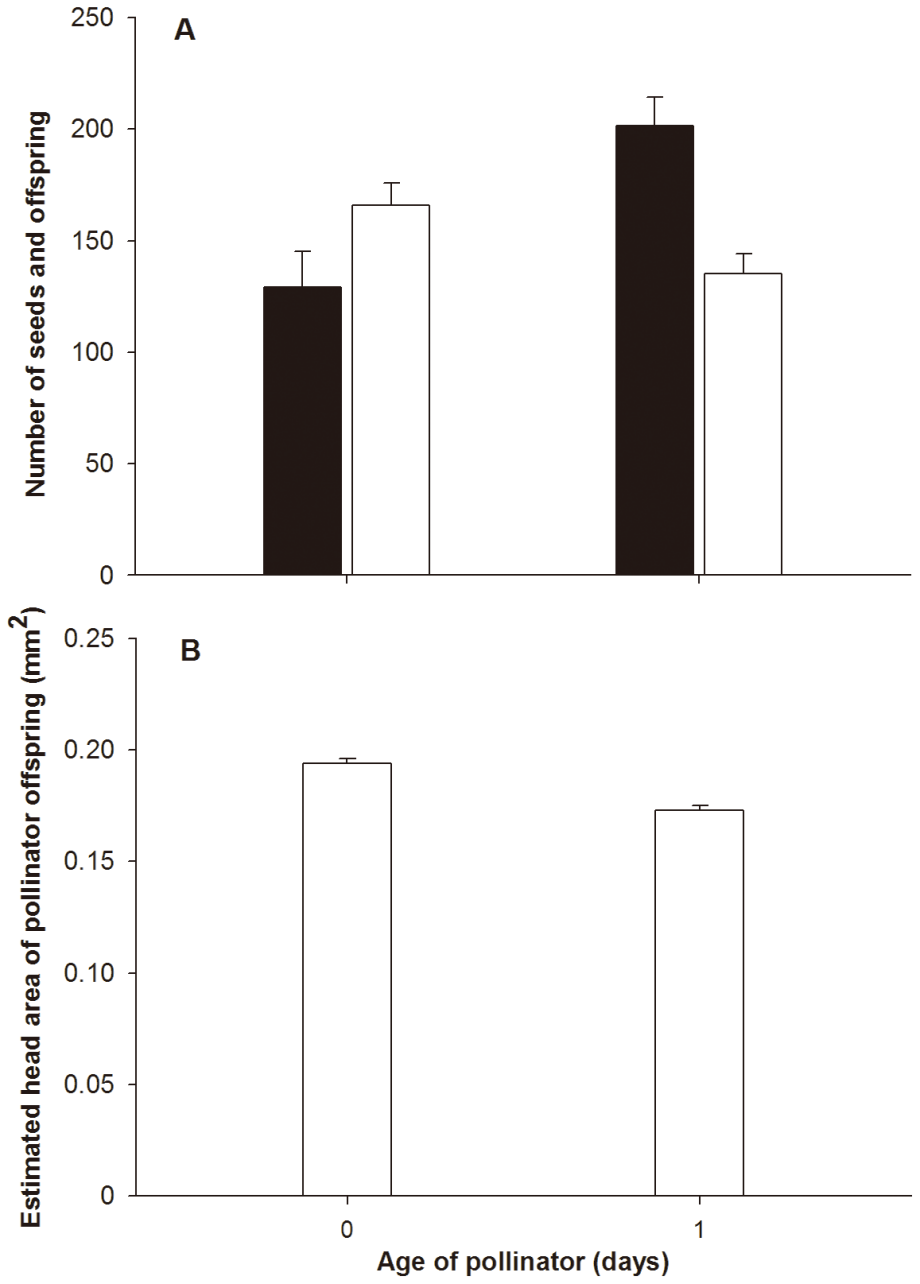
Seed and pollinator production in the figs that were entered by single pollinators of different ages. (a) Seeds (filled bars) and wasp offspring (open bars); (b) Offspring head sizes.

Single freshly-emerged pollinators that entered figs on the day that they had become accessible generated 129.08±16.21 seeds and 165.85±10.10 offspring (both sexes combined, n = 27 figs). The difference in seed and offspring numbers was not significant (paired-samples t-test: *t*_25_ = −1.72, *p = *0.10, Figure 3a). In contrast, when pollinators that had emerged the previous day entered the figs they generated 201.38±12.95 seeds and 135.24±8.75 offspring (n = 30 figs): significantly fewer offspring than seeds (paired-samples t-test: *t*_28_ = 4.40, *p*<0.001). Older pollinators also generated significantly fewer offspring than freshly emerged individuals, but larger numbers of seeds (ANOVA: *F*_1, 53_ = 5.29, *p*<0.05 and *F*_1, 53_ = 12.37, *p*<0.001 for offspring and seeds numbers respectively) (Figure 3a). Younger pollinators also produced significantly larger offspring than older pollinators (ANOVA: *F*_1, 198_ = 38.95, *p*<0.001) (Figure 3b).

### Experimental Introductions into *E*. *altissima* Figs of Varying Ages

**Figure 4:**
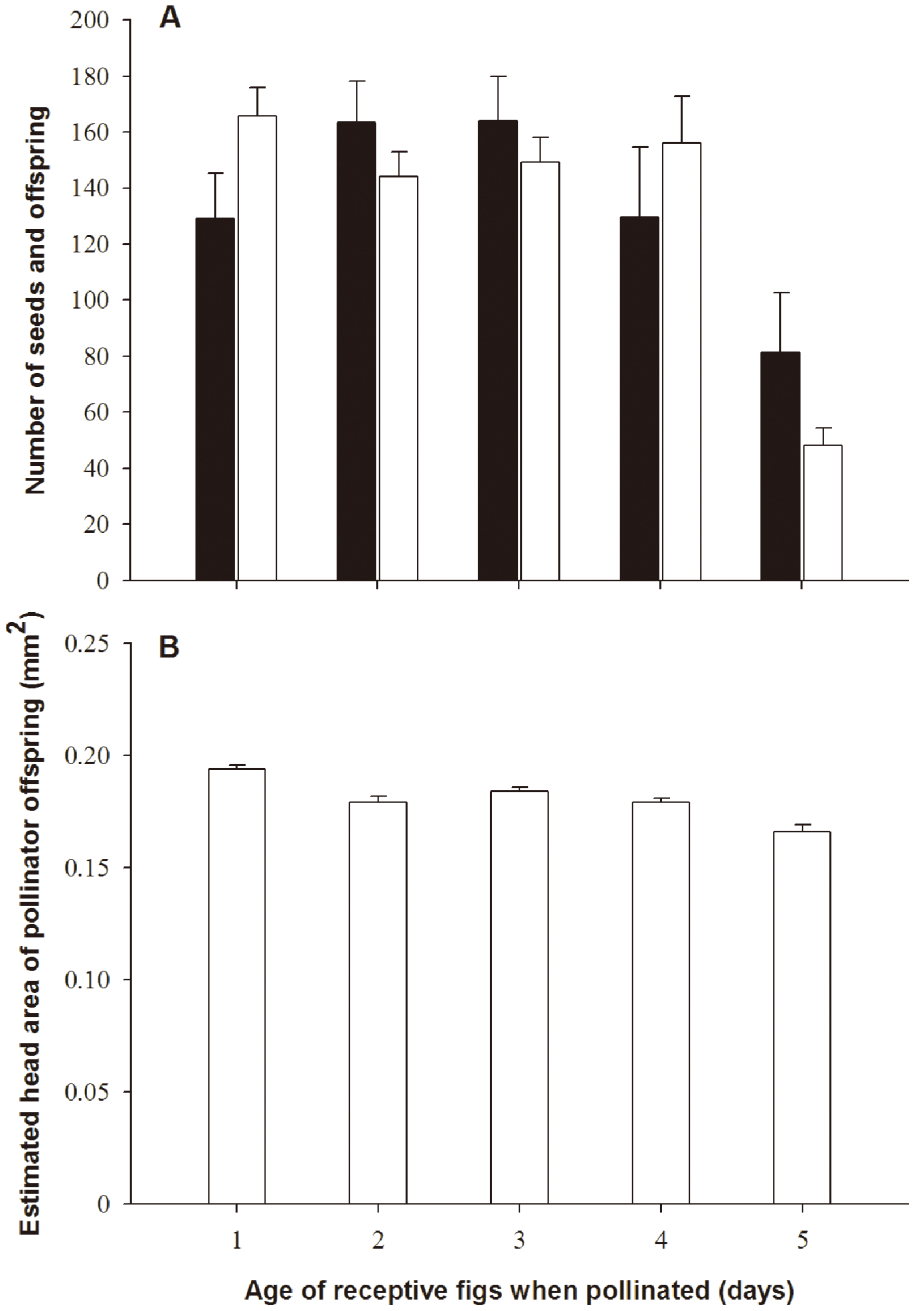
Seed and pollinator production in the figs that had been waiting for different lengths of time for fig wasps to enter. (a) Seeds (filled bars) and wasp offspring (open bars); (b) Offspring head sizes.

Un-entered figs of *F. altissima* remained attractive to female figs wasps for five days after becoming accessible, after which the fig wasps no longer attempted entry (Figure 4). Seed production peaked on the second and thirds days after the figs became accessible, whereas pollinator offspring numbers (both sexes combined) were stable until day four and then fell rapidly (Figure 4a). The numbers of both pollinator offspring and seeds therefore declined with fig age at entry (GLM: Poisson errors, β ± SE = −0.16±0.01, *z* = −27.83, *p*<0.0001; β ± SE = −0.09±0.01, *z* = −15.54, *p*<0.0001 for offspring and seeds production respectively, (n = 27, 25, 24, 23 and 21 figs for days 1–5 respectively, Figure 4a). Although seed production started to decline earlier than pollinator offspring numbers, across all five dates as a whole there was no significant difference in the numbers of offspring and seeds (paired-samples t-test: *t*_118_ = −0.02, *p = *0.98). The head size of female offspring also declined progressively with fig age at entry (LM: β ± SE = −0.01±0.001, *t* = −7.08, *p*<0.0001) (Figure 4b).

### Adult Female Longevity and Size

**Figure 5:**
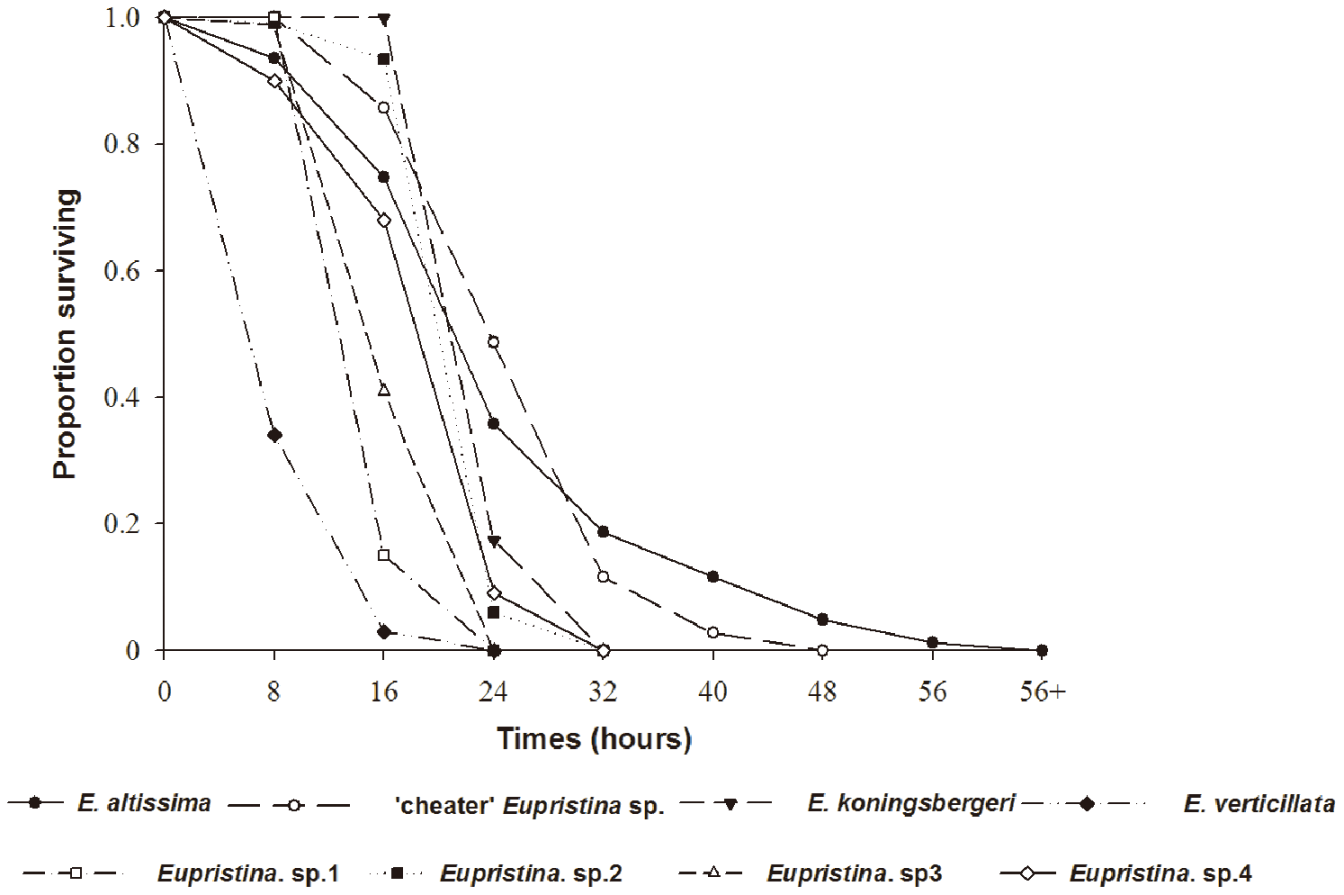
Survivorship of adult female *Eupristina* fig wasps maintained at 25°C.

Adult females of all eight *Eupristina* species died quickly when maintained at 25°C (Figure 5), but there were significant between-species differences in survival probability (Cox proportional hazards: β ± SE = 1.17±0.01, *z* = 15.79, *p*<0.0001). *E. verticillata* females died particularly quickly, with 97% mortality within 16 hours and 100% mortality after 24 hours. Adults of the two agaonids associated with *F*. *altissima* survived longest, though cheaters nonetheless only persisted at most for 40 hours and *E. altissima* had a maximum longevity of less than 56 hours.

**Figure 6:**
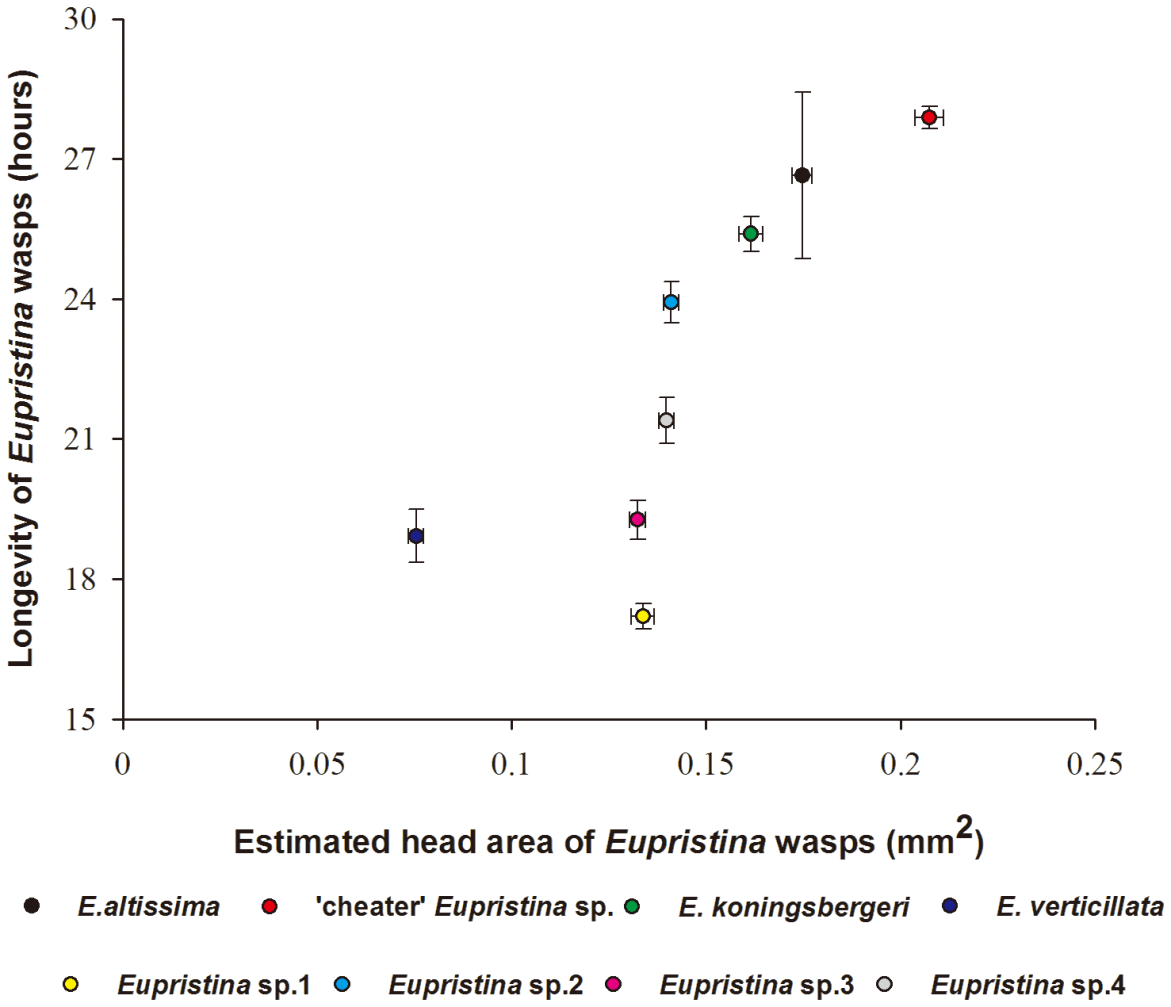
The relationship between longevity and body size in eight *Eupristina* fig wasp species.

The head sizes of the eight *Eupristina* varied considerably, with *E*. *verticillata* the smallest, and two agaonids associated with *F*. *altissima* the largest (Figure 6). Larger species lived significantly longer than smaller species (LM: β ± SE = 83.55±24.70, *t* = 3.38, *p*<0.05).

## Discussion

The pollination biology of *F*. *altissima* differs in detail from that recorded for any other *Ficus* species. Its persistent bract covers delay pollinator entry into figs that would otherwise be receptive and the tree’s pollinator fig wasp (and a non-pollinating cheater agaonid) are attracted to trees bearing figs before any of them are accessible, resulting in accumulations of fig wasps waiting for figs that they can enter. This contrasts with the typical relationship, where agaonids are attracted to their host trees by volatiles released from figs at the time when they are ready to be entered and pollinated. NPFW are often recorded waiting for figs to become suitable for oviposition [41], but in contrast to agaonids they are usually synovigenic, feed as adults and have adult longevities extending to several weeks [16].

Highly co-evolved interactions are often considered as model systems for the evolution of efficient chemical communication [42], but the de-coupling of pollinator attraction and fig accessibility in *F*. *altissima* appears to be contradict this assumption because de-coupling must inevitably result in increased mortalities among waiting pollinators, and also leads to pollination of figs by older fig wasps and of older figs being pollinated. It has been estimated that only around 1% of pollinators that depart from natal trees manage to find and enter typical receptive figs [20]. The delays brought about while the pollinator of *F*. *altissima* waits for figs to become accessible must inevitably increase pre-entry mortalities resulting from dehydration and predation. Their preference for resting on the underside of leaves may help reduce associated mortalities, but they make no effort to drink water droplets (Y Zhang, Pers. Obs.). The females are also relatively quiescent when sheltering under the leaves, with none of the intraspecific aggression displayed when they are competing to enter the figs.

Figs of *F*. *altissima* entered by pollinators that had emerged 24 hours previously contained more seeds, but fewer pollinator offspring than figs entered by recently-emerged foundresses. The balance between seed and pollinator offspring numbers also varied with the ages of the figs when they were entered, and older figs also generated smaller fig wasp offspring, as recorded previously in figs of another *Ficus* species [23]. There is reproductive conflict between pollinators and host fig trees that have a monoecious breeding system because the reproductive success of host trees is related to the numbers of seeds and pollen-carrying female fig wasp offspring that its figs produce, whereas the reproductive success of foundress females is linked only to the number of its offspring [20], [43]–[45]. The delay in access to *F*. *altissima* figs generated by its persistent bracts appears to favor relative female reproductive success for the tree (seed production) at the expense of its male reproductive success (pollinator offspring numbers), but with the added expense of reducing the total number of pollinators that enter its figs. Pollinator age variation and fig age variation are also likely to influence relative male and female reproductive success among fig trees in general, and may contribute to the stability of the mutualism.

Adult females of the two agaonids associated with *F*. *altissima* survived longer under our experimental conditions than the females of six congeneric species that pollinate other tree species at XTBG. Greater longevity is clearly advantageous to the species associated with *F. altissima*, because there is the chance that they will be attracted to a host tree that has no accessible figs when they arrive. However, they were also the two largest of the eight species we examined and larger insects are expected to be more desiccation resistant, because they have a smaller surface area for water loss relative to their weight [46]. Whether their relatively large body size is coincidental and pre-adaptive to the waiting imposed on them by their host plant, or an adaptive response, is unclear. However, the two species are not unusually large relative to the diameter of their host figs.

The adaptive significance of persistent bracts around the figs of *F*. *altissima*, and the associated delayed entry of its pollinators, remains to be established. One potential benefit from having persistent bracts may be greater protection of the plant’s ovules from NPFW that oviposit from the outside of the figs. Many fig trees support NPFW that lay their eggs during the earlier stages of fig development, but the persistent bracts of *F*. *altissima* prevent this.

Un-entered figs of *F*. *altissima* only remained receptive for five days after they became accessible. This is in contrast to the figs of a related monoecious species, *F*. *curtipes,* where some figs were found to still be receptive over 30 days after the onset of receptivity [47]. The apparently short period of receptivity displayed by *F*. *altissima* may be misleading, however, as it seems likely that the figs become receptive, and are emitting volatiles that attract their pollinators, several days before they become accessible to the fig wasps. Alternatively, the agaonids associated with *F*. *altissima* are perhaps responding to novel cues (perhaps related to the presence of young leaves) in addition to the attractant volatiles released from receptive figs.

In this study, we described an anomalous relationship between *F. altissima* and its wasps. Since *F. altissima* growing naturally in the forest are very tall, or if they are planted in a city they are easily influenced by human activity, it is difficult to closely observe the behavior of their tiny fig wasps. Only one tree at Xishuangbanna Tropical Botanical Garden was continuously observed 10 years, with the aid of a viewing platform, but the phenomenon of pollinators being attracted to inaccessible was regularly observed each year. Furthermore, the phenomenon was observed from a distance on other urban and forest fig wasp trees of this species. In conclusion, our results open an interesting and perplexing perspective on the co-evolution of this heavily-studied inter-specific interaction.
